# Accommodating heterogeneity in brand loyalty estimation: application to the U.S. beer retail market

**DOI:** 10.1057/s41270-022-00187-2

**Published:** 2022-10-14

**Authors:** Roozbeh Irani-Kermani, Edward C. Jaenicke, Ardalan Mirshani

**Affiliations:** 1 Fred Pirkle Engineering Technology Center, 440-C, P.O. Box 2088, Huntsville, TX 77341-2088 USA; 2 Pennsylvania State University, 208-B Armsby, University Park, PA 16802 USA; 3 The Pennsylvania State University, 291 Dorset Rd, Waban, MA 02468 USA

**Keywords:** Consumer choice models, State-dependent variables, Heterogeneity, Price imputation, Homescan data

## Abstract

The assessment of consumer behavior in brand-choice models may be greatly influenced by accurately modeling and evaluating a brand-loyalty parameter. The most well-known approaches for estimating brand loyalty employ a household’s past purchase data and account for habit formation with a single smoothing parameter that indicates the weight assigned to households’ current decisions versus their distant shopping history. In this study, we present a method for estimating time-varying smoothing parameters for heterogeneous households. We estimate smoothing parameters for American households from 2014 to 2017 using Nielsen Homescan data for the US beer retail market. Using this more flexible method, we discover that the smoothing parameter varies significantly among households and time frames (for the same households). We next include a brand-loyalty index based on this approach into a brand-choice model of the American beer retail market, demonstrating that this new method improves estimation results.

## Introduction

Brand loyalty, defined as a state-dependent persistence for a specific brand regardless of potential price changes, is essential for understanding consumers’ decision-making processes (Dubé et al. 2008). Brand loyalty is a critical marketing objective, as enterprises with a loyal customer base will enjoy a predictable revenue and a persistent demand (Seetharaman 2004). Brand loyalty may also act as a barrier to entry (Aaker 2009), increasing consumers’ willingness to pay (Schmalensee 1974; Aaker 2009), lowering consumers’ price sensitivity (Reichheld and Sasser 1996), and decreasing consumers’ drive to switch (Increasing the switching cost) (Dick and Basu 1994). Additionally, brand loyalty acts as a buffer, protecting businesses strategically from marketing clashes and offering necessary time for responding to rival maneuvers (Aaker 2009). Keane (1997) discusses the necessity of controlling for unobserved heterogeneity among households and state-dependent variables when estimating brand-choice models, both of which can be directly connected to brand loyalty. Chaudhuri and Holbrook (2001) investigate the relationship between loyalty and market share and relative pricing. Recently, scholars are investigating brand loyalty in e-commerce and online communities, and the findings indicate the importance of customer engagement and community characteristics that can influence brand loyalty (Chan et al. 2014; Hollebeek 2011). Briefly, brand loyalty is a strategic asset for a company’s products that may be earned through long-term marketing efforts or consumer personal experiences (Kotler et al. 2000), and must be maintained throughout the product’s life cycle (Bennett and Rundel-Thiele 2005).

The origins of brand loyalty lie in the consumer’s optimization dilemma. Families might save time by skipping the decision-making process, repurchasing the same brand regularly, and establishing a purchasing routine (Howard and Sheth 1969). This occurrence is more likely for frequently purchased products, remarkably inexpensive ones with lower switching costs. In this approach, a previously preferred brand is more likely to be picked again. This behavior is a true example of positive state dependency, or brand inertia, as it is labeled under such circumstances. Scholars discussed strategies for differentiating brand loyalty and inertia and developed definitions and assessment techniques to aid them in this process (Dick and Basu 1994; Reichheld 2003; Jones and Taylor 2007; Russell-Bennett et al. 2009).

Solving the consumer’s purchasing problems in such a manner is based on situational factors. Households’ purchase histories can reveal a lot about their future behavior. In recent years, marketing practitioners, behavioral economists, researchers, and data scientists have been working to find ways to understand households’ decision-making better and predict their possible future decisions based on their past purchase histories (Worthington et al. 2010). The bulk of these studies suggest that past experiences with brands affect households’ desires to consume the same or different brands in the future, and when the effects are significant, there is structural state dependency in households’ brand choices over time (Seetharaman 2004).

Guadagni and Little (1983) made the first attempt to incorporate brand loyalty as a part of a discrete choice model. As Seetharaman (2004) explains later, the most common way of modeling positive structural state dependency in the literature is yet based on an exponentially smoothed loyalty variable, and this modeling structure was first introduced by Guadagni and Little (1983). The potentially significant effects of these state-dependent variables motivate marketers’ employment of promotional schemes to create loyalty and balance the promotional costs with long-term profit (Seetharaman 2004). Brand loyalty is not a trick or just a managerial figure of speech. Now that it is measurable in different ways, major manufacturers like Del Monte, Harley Davidson, and General Motors spend considerable amounts to increase customer loyalty (Alonzo 1994; Lefton 1993; Helgesen 2006). Accurately modeling and measuring a brand-loyalty parameter can drastically affect the measurement of consumer impacts and welfare in brand-choice models. It has been an essential concept in economics and marketing literature for the past few decades (Cengiz and Akdemir-Cengiz 2016). Chaudhuri and Holbrook (2001) examine the influences of loyalty on market share and relative price.

To calculate structural state dependence, researchers defined loyalty as a weighted average of past purchase history based on a single smoothing parameter representing the weight a decision-maker gave to their recent experiences compared to their distant shopping history. This smoothing parameter was identical for all households. Researchers have improved the accurate calculation of that single smoothing parameter (Guadagni and Little 1983; Fader et al. 1992), but not considering the smoothing parameter as a varying factor over time and across households is similar to an assumption about static behavior among consumers who are changing daily and causes limitations in controlling for heterogeneity. In this study, we propose a model that considers the smoothing parameter a household-specific element that may change over time due to demographical, environmental, or geographical changes.

## Literature review

A brand is a distinguished name or symbol which is intended to identify a seller (or their product/service) to differentiate them from competitors (Aaker 2009). Customers might trust brands as facilitators who would filter the market on their behalf to make the decision-making process easier (Sullivan 1998). In other words, as defined by the American Marketing Association, a brand is “a name, term, sign, symbol, or design, or a combination of them, intended to identify the goods or services of one seller or group of sellers and to differentiate them from those of competitors.” The emphasis on differentiation in all these definitions shows how crucial it is for brands to help and protect their customers in competitive markets. Brands represent characteristics that differentiate products and services that, at the first glance, are designed to satisfy similar needs. These features could be functional, rational, or tangible for quantifiable product/service attributes, or they could be symbolic, emotional, or intangible for more abstract characteristics (Kotler and Armstrong 2012). Recently and with technological advances, the tangible and functional characteristics often exceed what consumers need, which shifts the competition toward the intangible elements of brands Noble and Kumar (2008).

According to marketing literature, loyalty is a biased reaction that is persistent over time and evident in the historical patterns of an individual, a family, or a business unit Mellens et al. (1996). To differentiate between inertia and loyalty in purchasing patterns, Jacoby and Chestnut (1978) argue that a commitment factor is a necessary component of loyalty.

Jacoby and Chestnut (1978) define brand loyalty as a time-related, biased psychological reaction displayed over time by a decision-maker toward one or more alternative brands from a group of such brands. This definition is generally accepted in economics and marketing literature, and it encompasses the majority of brand loyalty issues. According to Jacoby and Chestnut (1978), brand loyalty requires that decision-makers demonstrate commitment to a brand. This final phrase of their definition underscores that the action is the consequence of a psychological process biased by this commitment. Therefore, although consistent repurchasing of a brand might be interpreted as brand loyalty, it is not identical to inertia. Inertia is defined as a repeated brand choice not based on commitment but rather because the consumer is unwilling or unable to spend time and energy to find other brands to fulfill his desires better (Mellens et al. 1996). Thus, brand choice based on inertia has related but different motives and decision rules and may require different marketing strategies compared to brand choice based exclusively on loyalty (Hoyer 1984). Consumers who purchase products based on inertia usually stick to some simple rules for the decision-making process. In contrast, what influences brand loyalty are distinctive characteristics, design features, or a brand’s social image (Riezebos 1994).

Throughout the years, other scholars have somewhat adjusted this definition (Assael 1984; Mowen 2000; Wilkie 1994; McAlister and Pesemier 1982). Based on two essential characteristics, empirical methodologies for assessing brand loyalty may be categorized into five types. The first and second categories are concerned with measuring techniques, which might be brand- or individual-based. The third and fourth categories indicate whether the assessment relies on revealed preferences (behavioral) or on stated preferences (attitudinal) (Mellens et al. 1996). Additionally, one may build a fifth category in which scientists have combined attitudinal and behavioral components (Raju et al. 1990; Cleff et al. 2018).

Behavioral loyalty focuses on purchasing patterns and the proportion of times a purchaser chooses the same product or service in a specific category compared to the total number of purchases made by the purchaser in that category defines customer loyalty (Rundle-Thiele and Bennett 2001). This approach will not identify the factors that are causing this repetition. In other words, the behavioral approach cannot tell if the repetition is because of customers’ willingness to pay more for the brand or the attributes (Jacoby and Chestnut 1978; Dick and Basu 1994). Throughout the last several decades, several researchers who have critiqued the behavioral approach have highlighted that the notion of customer loyalty must incorporate attitudes (Jones and Taylor 2007; Russell-Bennett et al. 2009). The attitudinal approach emphasizes psychological commitment (Mellens et al. 1996; Russell-Bennett et al. 2009), emotional connection (Jones and Taylor 2007), and a desire for recommendation (Reichheld 2003) as the reasons for these repeated purchases. This way, the attitudinal approach clarifies why individuals choose a specific product or service when it is not simply brand inertia. As these approaches were not significantly capable of providing accurate predictions for customers’ actions in the future (Mellens et al. 1996; Kumar and Shah 2004), a more reliable combined approach was proposed (Jones and Taylor 2007), which can combine both behavioral and attitudinal components of loyalty and provide a more accurate prediction.

With interest from economists, psychologists, and marketing specialists, a wide range of research exists on how state-dependent variables affect choices, enter the decision-making process, and can be modeled and parameterized (Winer and Neslin 2014). The majority of these studies examine how one brand competes with other consumer brand choices within a certain product category (Bentz and Merunka 2000). Ballantyne et al. (2006) claim that brands are no longer seen to be just representations of items with distinct qualities; instead, they are now viewed as encapsulating factors that represent the unique traits, characteristics, and lifestyle signals in customers’ community and their environs. Kato (2021) explains a similar term as a brand concept, which is defined as abstract notions that often stem from a firm’s efforts to differentiate itself from rivals (Park et al. 1991). As a result, behavioral economists have demonstrated an increasing interest in brand-choice research. Prior to this current interest, a brand-choice researcher’s primary objective was to determine the influence of marketing mix elements on the decision-making process of a customer (Bentz and Merunka 2000; Chib et al. 2004). These marketing mix elements, on the other hand, were product-related, not customer-related. Due to the significance of customer behavior, this omission prompted brand-choice modelers to incorporate household characteristics to account for heterogeneity and state dependence in order to fully account for a diverse set of factors influencing the consumer’s decision (Keane 1997; Seetharaman 2004; Dubé et al. 2010). When discrete choice modeling gained popularity in marketing research, researchers began constructing more precise models. The Generalized Method of Moments (Nevo 2000), Bayesian estimation (Jiang et al. 2009; Zenetti and Otter 2014), or Maximum Likelihood (Park and Gupta 2009) are all methods for estimating discrete choice models with preference heterogeneity from aggregate data. All of these models address the issue of price endogeneity, which is a side effect of dealing with revealed preference data and a source of contention in marketing research (Villas-Boas and Winer 1999; Chintagunta et al. 2005; Petrin and Train 2010). While addressing the endogeneity issue arising from the pricing strategy, Anderson and Kumar (2007) demonstrate that in the long run and in a more concentrated market, it would be optimal for a firm with a larger loyal base to invest in attracting more loyal customers by lowering the average price and running promotions more frequently to weaken the competition.

Since Guadagni and Little (1983) showed how a brand-loyalty index could improve customer choice models fit and increase the explanatory power of such models, other researchers have explored different paths to improve the index (Fader et al. 1992; Fader and Lattin 1993; Ortmeyer et al. 1991; Dong and Stewart 2012). The basic brand-loyalty index would define brand loyalty as the state dependence persistence for a specific brand regardless of potential price changes, which was calculated based on an exponentially weighted sequence of past purchases. Brand loyalty, according to their model, may account for two major effects: state dependence and heterogeneity. While their index captured changes in purchasing behavior, it did not differentiate between the sources of variance. It was impossible to determine which aspects of behavior were a result of heterogeneity and which were a result of changes within a household over time. Another restriction of their model was the inaccuracy in measuring the “smoothing” parameter that was the base for generating the brand-loyalty index. To address this shortcoming, Fader et al. (1992) developed a linear approximation of the loyalty index based on the Taylor expansion. While this technique partially overcomes the smoothing parameter measurement issue, it does so by relying exclusively on a single smoothing parameter for all households during the course of the inquiry. This method ignores the smoothing parameter’s influence on time-related variables. Thus, the smoothing parameter is not only constant across all households but also time-invariant. Using a Dirichlet-Multinomial model Fader and Lattin (1993) introduced another index that was capable of accounting for abrupt preference changes among households. Dong and Stewart (2012) integrate heterogeneity into this Dirichlet-Multinomial model. Researchers assess brand loyalty in more recent studies employing Bayesian models that rely on the same classic model and, as a result, utilize the same smoothing parameter in their models for capturing brand loyalty across households (Seetharaman 2004; Dubé et al. 2008, 2010). While these latest research employ state-of-the-art mixed multinomial logit models coupled with Hierarchical Bayesian techniques, the assessment of brand loyalty remains based on the same basic framework.

In this study, we present a method to improve the estimation of the smoothing parameters. This new parameter would help control heterogeneity by varying through time and across households and overcomes the limitations of estimating structural state dependency using a constant smoothing parameter which is widely common in the literature (Guadagni and Little 1983; Fader et al. 1992; Seetharaman 2004; Dubé et al. 2008, 2010). This technique incorporates demographic and purchasing data at the household level, revealing that the estimated brand-loyalty smoothing parameter varies significantly among households and across time. We next demonstrate that using this brand-loyalty variable improves the estimate of a brand-choice model for the United States beer retail market using the Akaike information criterion (AIC) and Bayesian information criterion (BIC).

## Model

We use the alternative specific mixed multinomial logit model (McFadden and Train 2000), commonly known as the random-parameters logit model (Cameron and Trivedi 2005) or logit kernel model (Ben-Akiva et al. 2001), to model brand preference in the retail beer market in the United States. According to this model, households’ choice probabilities are associated to a linear combination of the alternative variant and alternative invariant attributes:

1 $$\begin{aligned} P_{ijt}=\int _{-\infty }^{\infty }\frac{e^{V_{ijt}}}{\sum _{k=1}^{J}e^{V_{ikt}}}f(\theta )d(\theta ), \end{aligned}$$

where $P_{ijt}$ = the probability that at purchase occasion *t*, household *i* selects alternative *j*; $V_{ijt}$ = the determinate portion of brand j’s utility at purchase occasion *t* for household *i*; $= \sum \Omega _rx_{ijt}^r+\sum \Gamma _rz_{it}^r$ , where $x_{ijt}^r$ = rth alternative variant variable (marketing mix) of both brand *j* and household *i* at purchase occasion *t*; $z_{it}^r$= rth alternative invariant variable of household *i* at purchase occasion *t*; $\Omega _r$ and $\Gamma _r$ = Coefficients to be estimated; $f(\theta )$= the density function of $\theta$ The mixed multinomial logit model can incorporate sets of alternative invariant variables like age, income, educational achievement, and occupation, as well as alternative variant variables like discount, color, and taste.

We estimate a brand-choice model that considers the primary factors of a household’s beer brand choice. A brand is defined as a manufacturer’s product identified by distinctive characteristics. As a result, a brand may be offered in various containers with a variety of different UPCs while maintaining the same product qualities. For example, Coors Light is treated differently in this model than Coors Pure Light, but Coors Light is treated the same regardless of whether a customer purchases a bottle or a can. The marketing literature established the existence of some forms of state-dependent utility in choice models, particularly for packaged goods (Dubé et al. 2008). Several studies have found how newly purchased brands boost a brand’s utility for a decision-maker (Erdem 1996; Seetharaman et al. 1999; Horsky et al. 2006). To prevent magnifying the effect of state dependency and to control for the heterogeneity among households, we follow Keane (1997). However, we explicitly account for heterogeneity in the computation of the brand-loyalty index. Thus, we specify the consumption utility of brand j at purchase occasion t for household i as follows:

2 $$\begin{aligned} U_{ijt} =\alpha _{ij} +\Gamma Z_{it} +\Omega X_{ijt} +L_{(\lambda _{it},Y_{ijt})} +\xi _{jt} +\epsilon _{ijt}, \end{aligned}$$

where $$\alpha _{ij}$$ = Brand *j*’s direct contribution to the consumption utility of household *i*; $$Z_{i}$$ = A vector of household characteristics that are alternative invariant like age, income, education, etc.; $$X_{ijt}$$ = A vector of marketing mix variables that are likely to be time variants. Variables like price, discount, and availability are more likely to change over time, while others like color, taste, or container are less likely to change; $$L(\lambda _{it},Y_{ijt})$$ = The brand-loyalty index based on a smoothing variable $$\lambda$$ and the past purchase history, $$Y_{ijt})$$; $$Y_{ij}$$ = A vector of 0’s and 1’s that shows the past purchase history of households *i*. If the *r*th element equals one, it means household *i* has purchased product *j* at time *r*; $$\xi _{jt}$$ = Unobserved product characteristics that may correlate with price; $$\epsilon _{ijt}$$ = An unobservable random brand- and the household-specific error term. This is an *iid* disturbance term.

We specify the loyalty variable for individual *i* for brand *j* at purchase occasion *t* as follows:

3 $$\begin{aligned} L_{(\lambda _{it},Y_{ijt})}=\lambda _{it} L_{ij(t-1)}+(1-\lambda _{it})Y_{ijt}. \end{aligned}$$

We define the loyalty index as a weighted average of past purchase history. In (3), the smoothing parameter $\lambda _{it}$ shows how a consumer (household) weighs her past opinion against her most recent experience. The parameter $\lambda$ gives the comparative effect of the last purchase. If it equals one, then the consumer’s brand loyalty is based only on her past experiences and is unaffected by her most recent purchase. In other words, her brand loyalty is a constant that was established at the outset and will neither diminish nor increase over time.

If $\lambda$ is equal to zero, on the other hand, it indicates that the consumer does not trust her prior judgment and instead depends on her most recent experience. Understanding the exact value of this smoothing parameter is, therefore, crucial for marketing scientists. As this state-dependent variable has a substantial impact on the estimation of brand-choice models, it becomes even more crucial to estimate the proper value of this smoothing parameter. Therefore, we consider $\lambda$ as a time-varying smoothing parameter that changes across households.

**Fig. 1:**
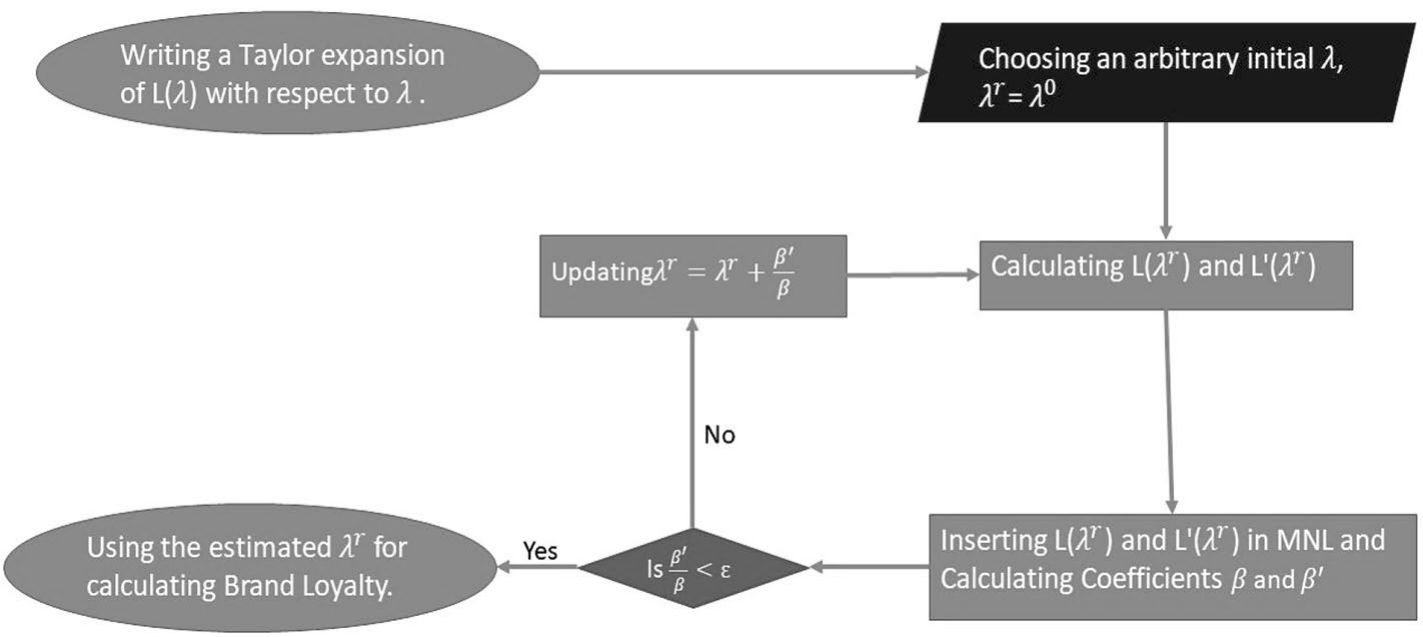
Smoothing parameter calculation algorithm

To estimate the time- and household-varying smoothing parameter, we make some methodological assumptions. Prior to determining the smoothing parameter, the technique requires a starting point (between zero and one). From here, we follow the procedure outlined in Fig. 1 which consists of the following five steps: (1) we prepare the data based on the previously described method for calculating the loyalty index based on an exponentially weighted sequence of past purchases. (2) We use a Taylor series expansion (Fader et al. 1992) where the exact formula for brand loyalty and its derivative with respect to the initial smoothing parameter is found using a recursive function. (3) We include these two calculated elements (brand loyalty and its derivative) and all the other variables in a Mixed Multinomial Logit model and recover estimated coefficients for both the brand loyalty and its derivative with respect to the smoothing parameter. (4) We then update the smoothing parameter based on the estimated coefficients. (5) As the last step, we repeat this process until the smoothing parameter converges, at which point we recover the estimated smoothing parameters for each household separately.

Using these estimated smoothing parameters, we can calculate the brand-loyalty index as a household-specific variable, add that to the Mixed Multinomial Logit model, and check for improvement in fit and explanatory power as the final step. To estimate $\lambda _{it}$ , we modified the non-linear estimation approach first introduced by Fader et al. (1992) to accommodate the heterogeneity in the smoothing parameter.

We expand $L(\lambda _{it},Y_{ijt})$ in a Taylor series around a starting value or initial point $\lambda ^0_{it}$:

4 $$\begin{aligned} L_{(\lambda _{it},Y_{ijt})}= & {} L_{(\lambda _{it}^0,Y_{ijt})}+\frac{dL_{(\lambda _{it}^0,Y_{ijt})}}{d\lambda _{it}}(\lambda _{it }-\lambda _{it}^0)\nonumber \\&+\sum _{n=2}^{\infty }\frac{d^nL_{(\lambda _{it}^0,Y_{ij(t-1})}}{d\lambda _{it}^n}\frac{\lambda _{it}-\lambda _{it}^{0}}{n!} \end{aligned}$$

If $L_{(\lambda _{it},Y_{ijt})}$ is a smooth function (i.e., its derivatives with respect to $\lambda _{it}$ are bounded) in an interval containing both $\lambda _{it}^0$ and the maximum likelihood estimate (MLE) value of $\lambda _{it}$, then the second- and higher-order terms in (4) will approach 0 as $\lambda _{it}^0$ approaches its MLE value.

If we now rename$\frac{dL_{(\lambda _{it},Y_{ijt})}}{d\lambda _{it}}$ as $L^{'}_{(\lambda _{it},Y_{ijt})}$ , we can rewrite the simplified estimation for $L_{(\lambda _{it},Y_{ijt})}$ as

5 $$\begin{aligned} L_{(\lambda _{it},Y_{ijt})} \approx L_{(\lambda _{it}^0,Y_{ijt})}+L^{'}_{(\lambda _{it}^0,Y_{ijt})}(\lambda _{it}-\lambda _{it}^0). \end{aligned}$$

This approximation will become exact when the second part converges to zero. Using a time-varying smoothing parameter that changes through time and among different households is the main characteristic of this model, which makes it different from the previous studies in this field.

In a rich data set, if we treat (5) as an equality and replace $L_{(\lambda _{it},Y_{ijt})}$ with its equivalent $L_{(\lambda _{it}^0,Y_{ijt})}$ + $L^{'}_{(\lambda _{it},Y_{ijt})}(\lambda _{it}-\lambda _{it}^0)$ within a conditional multinomial logit model which has (2) as the utility function, we can estimate the following coefficient, $\beta$, which determines $\lambda _{it}$ for each household separately:

6 $$\begin{aligned} \beta L_{(\lambda _{it},Y_{ijt})} \approx \beta L_{(\lambda _{it}^0,Y_{ijt})}+\beta L^{'}_{(\lambda _{it}^0,Y_{ijt})}(\lambda _{it}-\lambda _{it}^0). \end{aligned}$$

We then use (5) to replace $L_{(\lambda _{it},Y_{ijt})}$ in the conditional logit model where we observe two separate coefficients ($\beta$ and $\beta ^{'}$) for these new factors:

7 $$\begin{aligned} \beta L_{(\lambda _{it},Y_{ijt})} \approx \beta L_{(\lambda _{it}^0,Y_{ijt})}+\beta ^{'} L^{'}_{(\lambda _{it}^0,Y_{ijt})}, \end{aligned}$$

where $\beta ^{'}=\beta (\lambda _{it}-\lambda _{it}^0)$ means that at every step a new smoothing parameter for each household is calculated as

8 $$\begin{aligned} \lambda _{it}= \lambda _{it}^0+\frac{\beta ^{'}}{\beta }. \end{aligned}$$

To replace $L_{(\lambda _{it},Y_{ijt})}$ using (5) we need both $L_{(\lambda _{it}^0,Y_{ijt})}$ and$L^{'}_{(\lambda _{it}^0,Y_{ijt})}(\lambda _{it}-\lambda _{it}^0)$. Calculating the loyalty variable based on the initial smoothing parameter is possible using a recursive function similar to what we described in (3). If we rewrite (3), which is a recursive function based on its explicit formula, we get

9 $$\begin{aligned} L_{(\lambda _{it},Y_{ijt})}\approx & {} (\lambda _{it})^{(t-1)})(L_{ij(1)})\nonumber \\&+(1-\lambda _{it})\left( \sum _{s=0}^{(t-1)}(\lambda _{it})^s. Y_{ij(t-s)}\right). \end{aligned}$$

Using (9) as a general form of (3), we can now estimate brand loyalty for any household at any point in time.

The initial period, or starting point ($L_{ij(1)}$), presents another analytic problem that requires one more assumption: Based on the definition we accepted for the loyalty variable, we assume that at the starting point, the consumer has a similar brand loyalty toward all brands or

10 $$\begin{aligned} L_{ij(1)}=\frac{1}{n}, \end{aligned}$$

where *n* is the number of brands. While our initialization is different from Guadagni and Little (1983), it is in line with Fader et al. (1992). To estimate $L^{'}_{(\lambda _{it}^0,Y_{ijt})}$ we can now use (3) and find the first derivative of $L_{(\lambda _{it},Y_{ijt})}$ with respect to the smoothing parameter $\lambda$:

11 $$\begin{aligned} \frac{\delta L_{(\lambda _{it},Y_{ijt})}}{\delta \lambda }=\lambda _{it} \frac{\delta L_{ij(t-1)}}{\delta \lambda }+L_{ij(t-1)}-Y_{ijt}. \end{aligned}$$

Using (11), we can calculate the derivative based on all these known elements. Equation (11) is similar to (3) in structure, and both are computable recursively based on the initial conditions and the available data. Thus, we can compute a smoothing parameter for each household and each period. With the smoothing parameters calculated, we are now armed with an accurate brand-loyalty index, which we can then use in a brand-choice MML.

Based on (2), endogeneity arises when the unobserved $\xi _{it}$ correlate with price. We use the control function approach by Petrin and Train (2010) to control this potential price endogeneity bias. To implement the correction, we first regress observed brand prices on our model’s explanatory variables and instrumental variables. Then, in the next stage, we use the residuals from the first-stage pricing regressions as proxies for any unobserved factors in each brand’s demand that may be correlated with the respective brand’s price. Here, we use two Hausman instruments, i.e., the mean of prices of the purchased brand and other brands in markets from an outside region and the whole nation (excluding the target state) at the same time period.

## Data

This study uses Nielsen Homescan panel data on alcohol and cigarette purchases. Our sample spans the years 2014 to 2017 (3 years). Nielsen selects a geographically and demographically representative sample of US households. Households use a home scanner to keep track of purchases of food and related commodities, including the quantity purchased, the amount paid, and the date. Purchases consumed before returning home are often not scanned and are not included in the data set. Einav et al. (2008) evaluate the Homescan data’s reliability but highlight that its flaws and inaccuracies are on a par with those of many other collected data sets. The Homescan data set contains extensive information on each transaction and a wide variety of demographic statistics on the participating households. The data span 2014–2017 (3 years) and include transactions for over 2700 distinct beer brands.

Since we began this investigation, the United States has been struck by COVID-19, and the pandemic has also affected the retail beer sector. The consumption of beer off-premises has increased significantly (30%) (Lee et al. 2021). Consumers have shifted from trying new brands to purchasing the ones they already know and trust. Older and more well-known brands have had a sales growth revival. Significantly higher demand for cans, as opposed to bottles, is another practical difference (NBWA 2021).

**Fig. 2:**
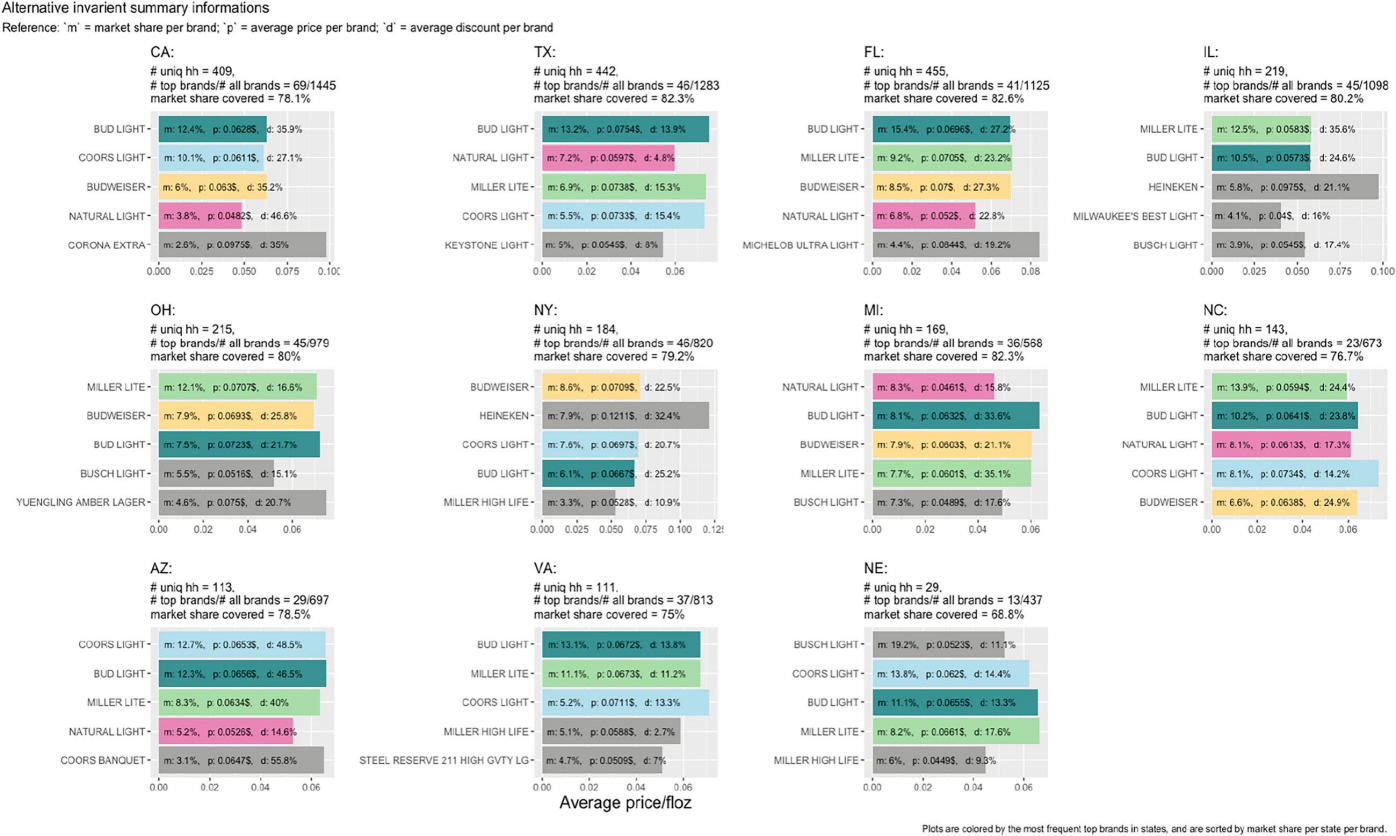
Alternatives variant variables for top five brands in different states

Despite the wide variety of brands, when states’ markets and brand market shares are considered, around 80% of transactions are tied to the top 2% of brands in each state when sorted by market shares. In this study, we examine eleven states from different regions of the United States that have a higher beer consumption rate. From 2014 to 2017 (3 years), these top eleven states consumed 60% of all beer consumed in the United States. Because the top 2% of brands vary by state, we study each state independently. Figure 2 shows each state’s top five bestseller brands during this study based on the defined data set discussed above. As we can see in this figure, the number of frequent buyers, beer brands (alternatives), and how the top 2% of the brands cover the market share vary in different states. Figure 2 also displays the top five brands’ price distribution in each of the eleven states with their market shares and average discount rates.

For computational convenience, we focus on these top 2% brands. If more than 90% of a household’s consumption portfolio is from the top 2% brands, we include that household in our data set. In discrete choice models, combining various alternatives into one outside option can distort parameter estimates and result in misleading predictions (Stafford 2018). Investigating more brands and covering each state separately enabled us to avoid using the outside option in the data set. Therefore, and for the scope of this study, we dismiss the outside option. This means the model can predict changes among consumers who chose the alternatives used in the model (around 80% of the market in each state) or conditional demand, but not in total demand because the model essentially does not contain any room to expand (Bonnet and Richards 2016).

We investigate households who purchased at least twelve times per year (regular purchasers) in each state. As expected, the number of regular purchasers is higher in more populated states. Prices, even for the same brands, change in different states. We can find similar beer brands and types standing among the top brands in all the states. Bud Light (colored dark green in Fig. 2) is consistently among the top five brands. Natural light (colored pink in Fig. 2) has a relatively lower price range among other top brands in all the investigated states. Among the imported brands, only Corona Extra and Heineken made the top five brands in some states.

For the mixed logit model used to estimate the smoothing parameter, we need the full vector of each consumer’s prices, containers, and discounts available on each purchase occasion for all brands in the choice set. However, the Homescan data set records these variables for the purchased brand. We follow a Bayesian approach for data imputation to overcome this empirical challenge. To impute each brand’s missing data, we use a prior distribution based on all purchased brands in that county on the purchase date (which gives us the weights). For observations, we look at the purchased prices, discounts, and containers recorded for each brand in a time span around the purchase date. In the first step, we look at the county level and search for the missing brand information in 8 days. In other words, if the missing brand has been purchased 4 days prior or 4 days after the purchase occasion, we will use all the observed information in that time span to form the discrete distribution as the likelihood distribution.

If a brand has not been purchased in the mentioned 8-day period, we expand the geographical area (from county to state) and the time span (from 4 to 7 days). Following the same pattern, we expand the geographical area to region and then to the total sample and the time span to 15 days and 6 months, if needed.

In addition to some household characteristics collected directly from Homescan, we construct other transaction variables based on the Homescan purchase data. We record each brand’s price and construct variables related to beer type (or flavor), purchased volume, amount purchased on promotion, product container information, and ultimately a brand-loyalty variable.

**Table 1:** Household characteristics

Characteristic	AZ, *N* = 95^a^	CA, *N* = 367^a^	FL, *N* = 382^a^	IL, *N* = 176^a^	Ml, *N* = 139^a^	NC, *N* = 106^a^	NE, *N* = 17^a^	NY, *N* = 163^a^	OH, *N* = 167^a^	TX, *N* = 390^a^	VA, *N* = 85^a^	*p* value^b^
Household income	58.89	67.39	56.57	61.39	58.02	54.33	59.94	60.25	61.77	68.79	73.44	0.003
	(40.43)	(44.75)	(37.98)	(41.05)	(38.21)	(33.93)	(40.51)	(41.64)	(41.08)	(43.18)	(49.05)	
Household size	2.47	2.35	2.35	2.43	2.43	2.39	2.06	2.38	2.49	2.49	2.60	0.2
	(1.16)	(1.28)	(1.15)	(1.20)	(0.96)	(1.07)	(0.75)	(1.27)	(1.27)	(1.21)	(1.24)	
Male head age	54.83	48.69	54.22	50.30	52.07	43.51	47.59	47.71	50.23	49.47	54.16	0.001
	(22.22)	(24.56)	(21.99)	(24.89)	(21.37)	(25.80)	(28.04)	(24.07)	(23.75)	(23.41)	(17.48)	
Female head age	54.06	43.96	46.31	48.89	51.27	51.80	58.76	46.61	50.02	49.79	49.40	0.001
	(20.74)	(25.19)	(24.69)	(23.83)	(18.28)	(19.50)	(17.15)	(23.84)	(21.37)	(21.14)	(21.13)	
Male head education	0.52	0.60	0.52	0.41	0.43	0.37	0.53	0.45	0.51	0.53	0.55	< 0.001
	(0.50)	(0.49)	(0.50)	(0.49)	(0.50)	(0.48)	(0.51)	(0.50)	(0.50)	(0.50)	(0.50)	
Female head education	0.58	0.57	0.51	0.51	0.47	0.60	0.59	0.43	0.44	0.56	0.56	0.013
	(0.50)	(0.50)	(0.50)	(0.50)	(0.50)	(0.49)	(0.51)	(0.50)	(0.50)	(0.50)	(0.50)	
Married	0.86	0.63	0.72	0.69	0.83	0.71	0.65	0.63	0.75	0.77	0.81	< 0.001
	(0.35)	(0.48)	(0.45)	(0.46)	(0.38)	(0.46)	(0.49)	(0.48)	(0.44)	(0.42)	(0.39)	
Hispanic	0.05	0.20	0.09	0.03	0.06	0.05	0.18	0.07	0.03	0.17	0.05	< 0.001
	(0.22)	(0.40)	(0.29)	(0.17)	(0.23)	(0.21)	(0.39)	(0.25)	(0.17)	(0.38)	(0.21)	

(Fe)Male head education: ’some college or higher’ $=$ 1 & otherwise $=$ 0. Married: married $=$ 1 & not married $=$ 0

^a^Statistics presented: Mean (SD)

^b^Statistical tests performed: Kruskal–Wallis test

Hispanic: Hispanic $=$ 1 & not Hispanic $=$ 0

Table 1 shows household characteristics across different states in the data set. These are the main alternative invariant variables we investigate in this study. To make sure these variables are significantly different across states, we executed a non-parametric Kruskal–Wallis test. For most of these alternative invariant variables (except for the household size), the *p* values in the last column show that not all the states medians are equal.

## Results

### Brand-loyalty index

**Fig. 3:**
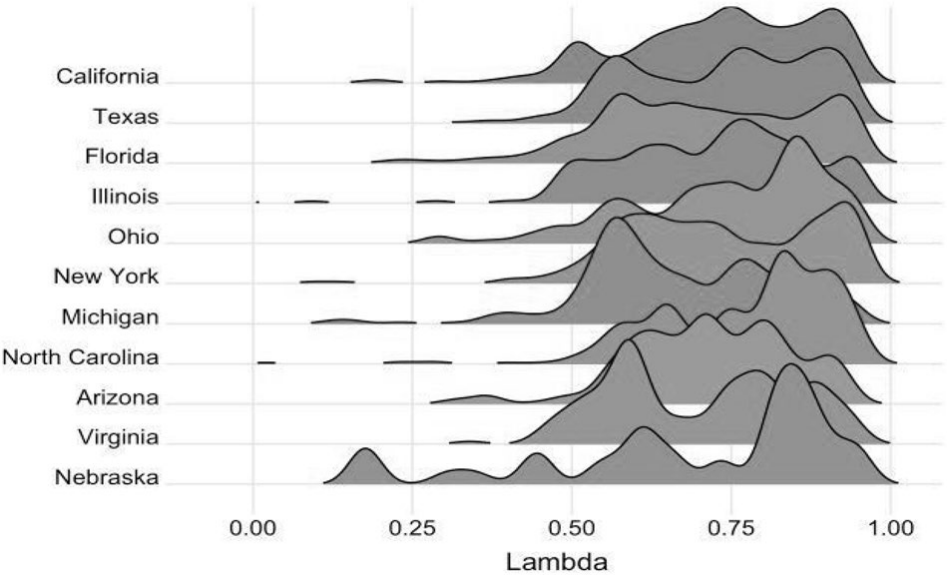
Distribution of the smoothing parameter across the states

To illustrate how our technique generates smoothing parameters that vary across households and over time, we developed a graph displaying lambda distribution in different states. Figure 3 shows the distribution of the smoothing parameters across different states. Smaller smoothing parameter magnitudes imply that a household prioritizes recent experience above past opinions. For all households, the smoothing parameter’s average value is 0. 72; yet Fig. 3 shows that individual households might deviate significantly from this average value.

### Brand choice

To demonstrate the relevance of heterogeneous smoothing parameters, we compare the new proposed brand-choice model with varying smoothing parameters and an alternative model with the classic, constant smoothing parameter. We build two separate models using beer retail market data from 2014 to 2017 (3 years). The preferred model calculates each household’s brand loyalty for each brand on each purchase date using the newly estimated smoothing parameters and incorporates this index into the MML brand-choice model. We compare this preferred model to a restricted model that incorporates a loyalty index that relies on a constant smoothing parameter of 0. 75 across different states. To account for potential price endogeneity, both the preferred and restricted models also include the estimated residuals from the auxiliary price equation that is part of the control function approach (Petrin and Train 2010). For the preferred model (Model I), Table 2 shows the parameter estimates for the alternative variant variables and the value of the log-likelihood function of both the preferred model and the restricted version (Model II). The results vary by state, and as expected, many of these alternative invariant variables are significant at the 1% level, including household size, income, age, and education, among others. The effects of these variables on the probabilities vary among the brands.

**Table 2:** Results for alternative variant variables across different states

Variables/states		CA	TX	FL	IL	OH	NY	MI	NC	AZ	VA	NE
Discount percentage	Coefficient	− 0.05	0.08	− 0.01	0.15**	− 0.12*	− 0.36***	− 0 29	− 0 37	− 0 37	0.59***	− 0.41
	Standard deviation	(0.04)	(0.05)	(0.04)	(0.06)	(0.07)	(0.07)	(0.08)	(0.09)	(0.09)	(0.12)	(0.5)
	P.Value	[0.132]	[0.115]	[0.827]	[0.011]	[0.086]	[0]	[0]	[0]	[0]	[0]	[0.407]
Loyalty	Coefficient	6 97***	6.48***	6.46***	6.35***	6.25***	6.51***	6.59***	6.07***	6.15***	6.27***	4.81***
	Standard deviation	(0.04)	(0.04)	(0.04)	(0.07)	(0.06)	(0.07)	(0.08)	(0.09)	(0.12)	(0.11)	(0.43)
	P.Value	[0]	[0]	[0]	[0]	[0]	[0]	[0]	[0]	[0]	[0]	[0]
Price Per Floz	Coefficient	− 12 57***	− 11.92**	− 11.6**	− 49 52***	− 8 32	− 15 92***	− 10.8*	− 29.69*	− 41.88***	− 23.3*	− 80.29*
	Standard deviation	(2.86)	(5.07)	(4.62)	(6.85)	(3.17)	(5.37)	(6.18)	(15.69)	(10.45)	(13.11)	(44.28)
	P.Value	[0]	[0.019]	[0.012]	[0]	[0.009]	[0.003]	[0.081]	[0.058]	[0]	[0.076]	[0.07]
Residuals (Control function)	Coefficient	12.84***	15.19***	11.3**	50.19***	8.47***	16.36***	13.65**	35.12**	44	18.83	131***
	Standard deviation	(2.87)	(5.2)	(4.66)	(6.9)	(3.22)	(5.46)	(6.65)	(15.8)	(10.63)	(13.3)	(45.51)
	P.Value	[0]	[0.003]	[0.015]	[0]	[0.008]	[0.003]	[0.04]	[0.026]	[0]	[0.157]	[0.004]
Rel-AIC	Model I	35,707	33,827	32,272	13,673	14,850	15,886	8919	9329	5961	5445	640
	Model II	35,834	34,339	32,521	13,807	14,997	16,218	8933	9524	6003	5487	671
Rel-log	Model I	− 17,300	− 16,531	− 15,780	− 6499	− 7204	− 7569	− 4319	− 4479	− 2777	− 2564	− 267
Likelihood	Model II	− 17,363	− 16,786	− 15,905	− 6565	− 7278	− 7735	− 4327	− 4577	− 2799	− 2584	− 283

Asterisks: (*) indicate significance at a 10% level (**) indicates significance at a 5% level (***) indicates significance at a 1% level

Models I and II produce comparable results: both models emphasize the necessity of incorporating the brand-loyalty index alongside the marketing mix variables in the choice model. Additionally, we discover that the residual terms are significant, demonstrating the essential need for accounting for price endogeneity. The negative price coefficient is interpreted as a substitution effect among brands (Krishnamurthi and Raj 1988), which was validated by examining cross-price elasticity in both models. Significant and negative discount coefficients, which may appear surprising, might be viewed as a quality indicator. Perhaps customers perceive advertised brands to be less fresh or of poorer quality than non-promoted brands (Simonson et al. 1994; Jacobson and Obermiller 1990). The coefficient of interest on the brand-loyalty index is significant with a positive sign, and thus it increases the probability of the brand being chosen. While Models I and II lead to similar results, we next investigate in more detail the question of whether the new model performs better than the restricted version in a statistical sense. More specifically, we estimate the goodness-of-fit statistics for these two models. There are a variety of techniques that may be categorized primarily into two branches for determining whether a model is the best model or the simplest model. One would be resampling, and the other one is the probabilistic approach. In the resampling approach of model selection, data are resampled into train/test for several iterations, followed by training on train and assessment on a test. This approach selects the optimal model based on performance, not model complexity. In this method, performance and error are calculated using out-of-sample data. Probabilistic model selection, a statistical method that quantifies the quality of a model by estimating the Information Criterion (IC), is the other method utilized in this study. It employs a scoring system that uses a probability framework of log-likelihood of Maximum Likelihood Estimation (MLE) to select the best model. In the probabilistic method, the IC is a statistical measure that yields a score. The model with the lowest score loses the least amount of information and is regarded as the best. In this method, we learn about the performance of models using the MLE concept, and the number of parameters helps us determine the model’s complexity. Combining these factors will form a score that helps the investigator decide which model is the best. In other words, the computed score promotes models with a higher goodness-of-fit score while penalizing overly complicated ones. There are three typical statistical approaches for obtaining such a score: AIC (Akaike Information Criterion), BIC (Bayesian Information Criterion) from frequentist probability and Bayesian probability, and Minimum Description Length (MDL) derived from information theory. When models are fitted by AIC/BIC, there is a potential that over-fitting will occur when the probability is raised by adding more parameters. The penalty element is therefore included in the calculation.

Table 2 shows that Models I outperforms Model II. We focus primarily on the Akaike Information Criterion (AIC), which tests the new brand loyalty variable’s additive explanatory power and the log-likelihood. We find that the difference in both AIC and BIC provides strong support for Model I in all the investigated states.

**Fig. 4:**
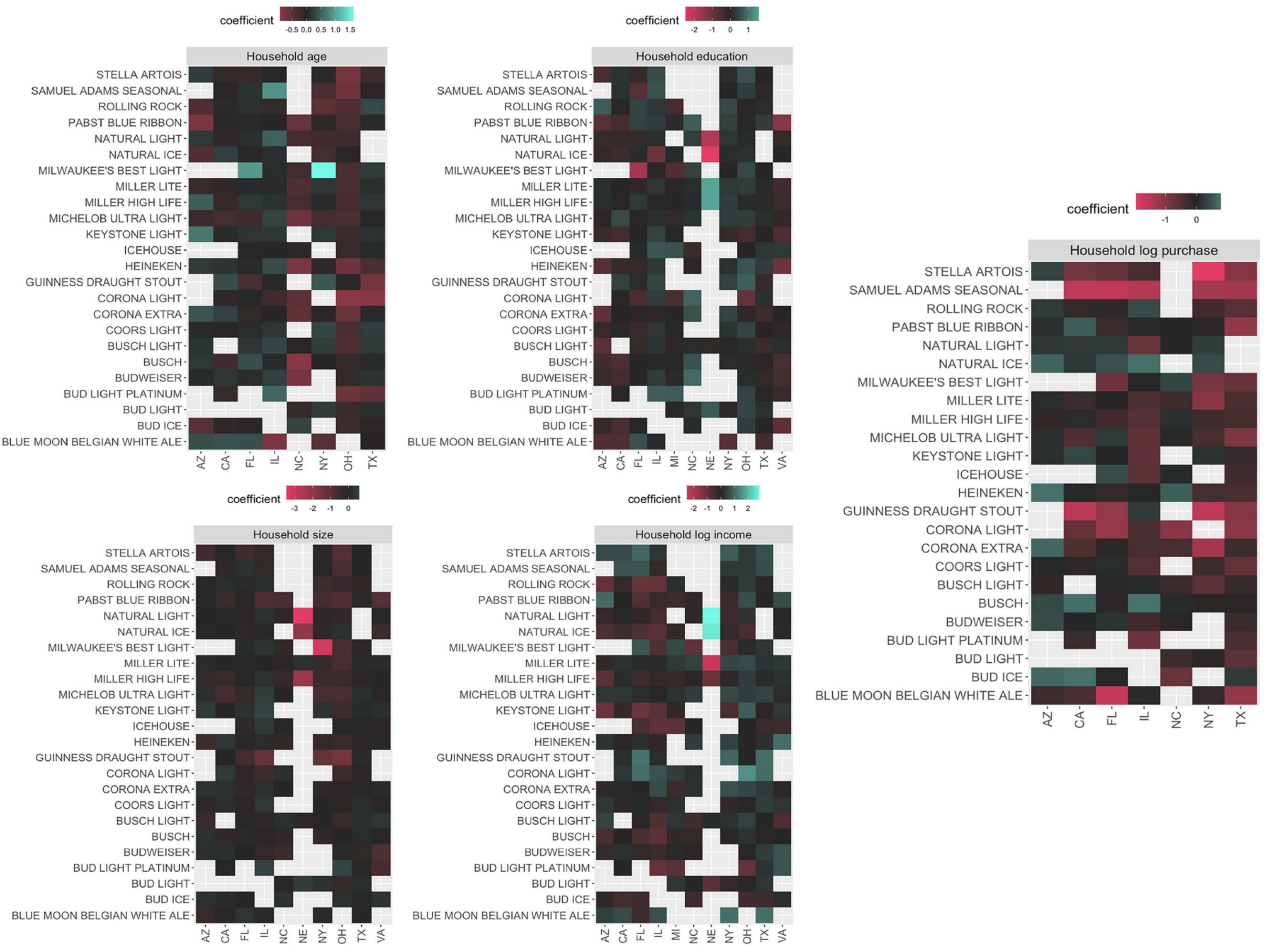
A visualized summary of alternative invariant variables coefficients

For the rest of the paper, our discussion focuses on Model I, the best-performing model based on the Akaike Information Criterion (AIC) and Bayesian Information Criterion (BIC), which test the additive explanatory power of the new brand-loyalty variable. Figure 4 presents a summary of results for alternative invariant variables across the eleven states for the top 24 best seller brands in the investigated states. Empty (gray) cells in Fig. 4 represent cases where the coefficients are not significant at the 1% level. According to our results, alternative invariant variables affect the likelihood of a purchase differently in different states. For example, while a higher education level would make it less likely for a household to choose the famous imported brand, Heineken, in California or Florida, a higher education level increases the likelihood of selecting that same brand in Texas and Illinois. Looking at the income effect, we observe how households are less likely to choose relatively cheaper brands when their income level increases, yet the results are not always consistent across all states for all brands. For example, while in five states (CA, FL, IL, NY, and AZ), an increase in income level makes it less likely for households to choose Keystone Light (a relatively cheap brand), in Ohio, households would select that brand more often when their income increases. Figure 4 also reveals how other variables like age, education, and household size affect brand choices differently in different states.

**Fig. 5:**
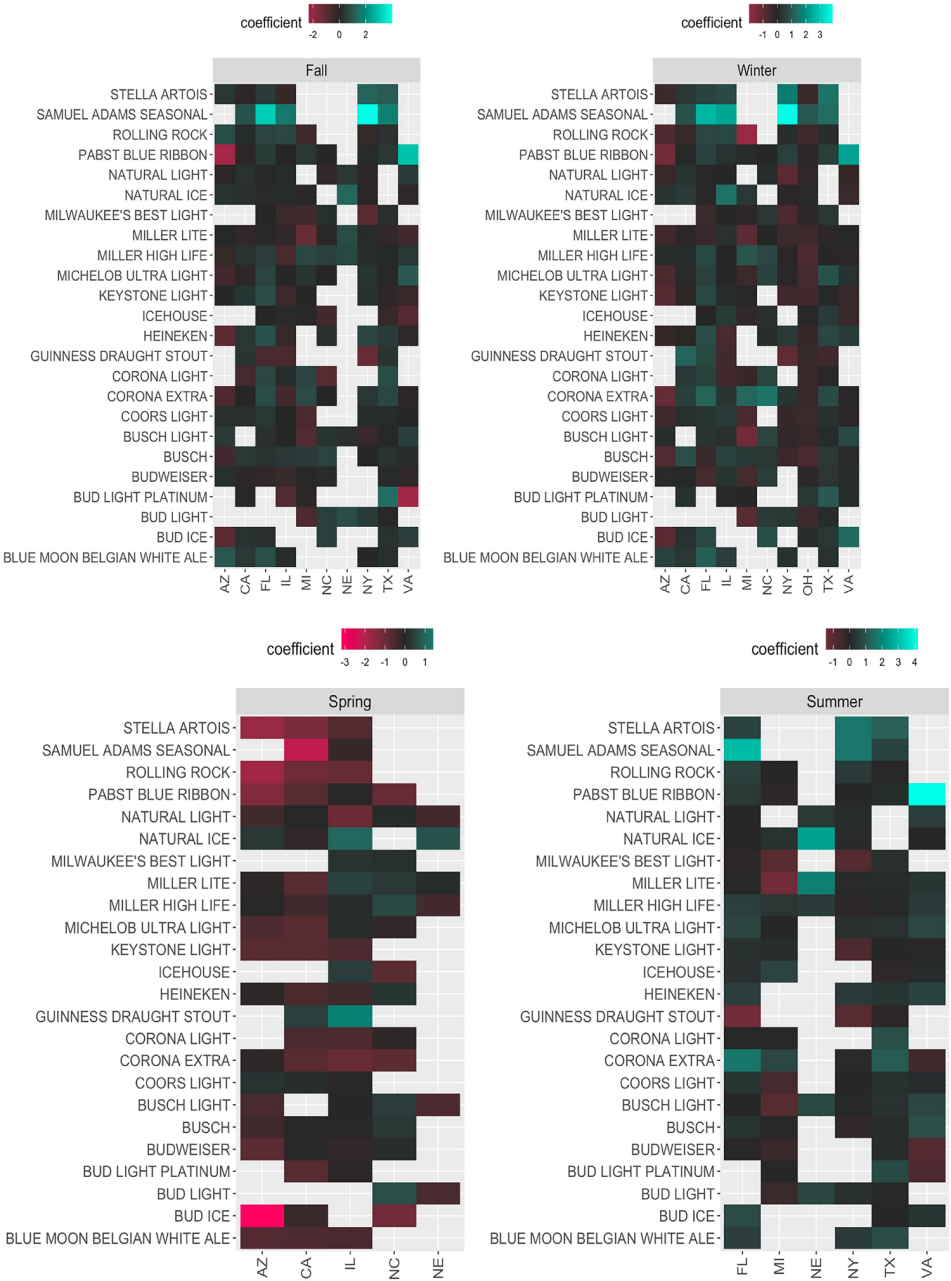
A visualized summary of seasonality (quarters coefficients)

The other factor which significantly affects brand choices is seasonality. Like other factors, seasonality works differently across states, even for similar brands. We can observe these differences by looking at the visualized summary of the seasonality effects in Fig. 5. For example, while purchasing beer in winter might increase the probability of picking Pabst Blue Ribbon in Virginia, it might decrease the probability of picking that same brand in Arizona. Seasonality does not have a similar impact on all brands; in many cases (Seasons/States), the coefficients are insignificant. Figure 5 also reveals how Winter and Fall affect brand consumption more than Spring and Summer as we have more significant coefficients in Winter and Fall than Spring and Summer across the states.

## Conclusion

We have demonstrated that increasing the heterogeneity of the smoothing parameter that serves as the basis for a brand-loyalty index is computationally viable and increases the explanatory power of an estimated brand-choice model. Accurately assessing and quantifying brand loyalty among households may benefit manufacturers and retailers primarily by enabling them to better target their client base and quantify the effects of brand loyalty. However, it may also benefit policymakers by allowing for more exact welfare estimation due to mergers that change brands’ availability.

Additionally, this model can be applied to other scenarios requiring a comparable degree of heterogeneity in state-dependent variables. For example, enhanced heterogeneity could improve political party polling or simplify risk assessment for insurers, banks, and lenders. Thus, a significant implication of our study is that it advocates for the inclusion of heterogeneity in different state-dependent variables in other empirical situations.

When we apply the model to the US beer retail market, we discover how brand loyalty has a significant impact on brand choices. Thus, having a precise measure of brand loyalty and tracking its progress over time will help firms enhance their segmentation and targeting strategies. Consistent with earlier research, we observe a significant effect of marketing mix factors.

By definition, the smoothing parameter describes how households weigh their experiences over time. By utilizing such a parameter, one may ascertain who will remember their feelings and who will forget. Here, more precise data provides marketers with a useful tool for segmentation, targeting, releasing a new product, or even rebranding.

Accurately measuring brand loyalty and analyzing trends in brand-loyalty variations will assist market planners in predicting demand, simulating segmentation, and formulating effective positioning strategies. In the digital era, tracking loyal consumers, discovering their purchase patterns, and making appropriate plans are now feasible. For example, to lunch new products, learning about customers’ brand loyalty in different regions and various markets would benefit the industry and help market analysts to optimize their approaches toward new markets. While identifying the model, this new time-varying smoothing parameter assisted us in achieving greater accuracy than previous models, albeit at a cost. Calculating these new smoothing parameters is computationally intensive and requires a significant amount of computing power, especially when working with big data. While consumer databases continue to grow in size, models must be adaptable and scalable (Braun and Damien 2016; Chintagunta et al. 2016; Bradlow et al. 2017). Increasing the investigation time, the number of brands, or the number of households would require greater processing power. However, assuming Moore’s law remains valid, computing technology continues to progress, and machine learning improves at the same rapid pace it did recently, this load will soon be alleviated (Jacobs et al. 2016).
